# A critical examination of ‘family’ caregiving at the end of life in contexts of homelessness: Key concepts and future considerations

**DOI:** 10.1177/26323524251336765

**Published:** 2025-05-13

**Authors:** Ashley Mollison, Kelli I. Stajduhar, Marilou Gagnon, Ryan McNeil

**Affiliations:** 1 Institute on Aging and Lifelong Health, University of Victoria, BC, Canada; 2 School of Nursing, University of Victoria, BC, Canada; 3 Medicine, Public Health and Anthropology, Yale University, New Haven, CT, USA

**Keywords:** family caregiving, informal caregiving, alternative family forms, nuclear family, fictive kinship, non-kin caregiving, equity, homelessness, palliative care

## Abstract

Identifying and addressing inequities in palliative care is an area of growing interest and importance. In this critical essay, we aim to challenge embedded assumptions about ‘family’ caregiving in white, Western systems (e.g. that of the nuclear family as carers) and focus on how the social determinants of health (SDOH; e.g. income and social protection, housing, education, food security) affect access to, and quality of, care at the end of life. More specifically, our analysis pays attention to what shapes the SDOH themselves including how racism, classism, heterosexism, and ableism become embedded and sustained in health and social institutions including palliative care. We begin by providing a brief discussion of the study of ‘family’ including the nuclear family standard and fictive kinship as an ‘alternative’ family form. Next, we focus on fictive kinship in two diverse populations – (1) street-involved youth who form street families; and (2) older adults who access care beyond nuclear families – that challenge embedded assumptions and help set a foundation for thinking about family and caregiving in contexts of inequities. Drawing on short vignettes, we then focus on emerging issues in palliative care and ‘family’ caregiving in contexts of homelessness and housing vulnerability. These issues include how caregivers in contexts of homelessness are, themselves, facing structural vulnerability; bio-legal family estrangement, reunification, and privileging; and how community service workers are filling both formal and informal caregiving roles. We conclude by delineating ongoing questions, research and practice gaps, and suggestions for future research in this area.

## Plain language summary

Looking at ‘family’ caregiving at the end of life among people who are homeless: Key issues and suggestions for future research

Society often expects that family will be around to help if you are sick. This is not the case for everyone. Sometimes people live far apart from their families. Sometimes family is not available or there are bad relations between family members. People get care in other ways, though, such as from friends, neighbours, and chosen family. In this essay, we look at two situations where people are getting care outside people they are related to. The first is homeless youth who form ‘street families’. The second is older adults who get care from friends, neighbours and others they think of as family. Next, we offer some stories based on past research to show what happens for caregivers when they are looking after people who are homeless. We finish by talking about some gaps and next steps for research in this important area.

## Introduction

Approximately 75%–90% of home-based care at the end of life is provided by informal caregivers, making informal caregiving central and essential to the provision of palliative care.^1^ While the benefits of informal caregiving are vast for the healthcare system, patients, and caregivers themselves, caregiving is socially, emotionally, physically, and financially demanding^2,3^ and intensifies at the end of life.^4,5^ Decades of activism, research, and ‘calls to action’ to improve the conditions of, and reduce burdens on, caregivers underscores how family caregiving is also an important equity issue.^1,6,7^ Indeed, caregiving is largely carried out by women, with poor and racialized women disproportionately experiencing the negative impacts of its demands.^7,8^ Beginning in the 1980s, neoliberal ideology featuring market-driven health and social policies have prompted shifts in responsibility of care from the state to individuals and families, intensifying inequities and vulnerabilities of caregivers.^6^ Today, social hierarchies are reproduced in caregiving as its financial and social costs further disadvantage those who are already socioeconomically disadvantaged, stratified across gender, race, and class lines.^1,9,10^ For palliative care to truly attend to the person with serious illness and their caregivers,^11^ we must understand informal caregiving within a global context of gendered and racialized, undervalued ‘care’ work^12^ and subject to economic, political, and social forces that impact how care is provided and received.

While healthcare systems the world over already rely heavily on informal family caregiving, demographic trends in the Global North point towards a greater reliance on informal caregivers in the future.^13,14^ These caregivers will be tasked with supporting people who are living longer with increasingly complex health and social needs.^4,13,14^ Yet, the capacity to take care of these needs ‘in house’ – within the nuclear family arrangements presumed by these systems – is dwindling.^15^ Smaller family sizes, greater distance between families, and family diversity in the Global North^16,17^ means that the ways in which people receive and provide care are changing.^15^ These changes in family structure, combined with a complex and ageing population, raises concerns about how those ageing alone and isolated will get their needs met as they require care in community settings.^18–20^ Yet, research also shows that older adults who are ageing ‘alone’ may expand their networks to include extended family, friends, neighbours, and community members^21–28^ who may be mobilized for care at the end of life.^29^ For palliative care to continue supporting families and caregivers alongside patients, there is a need to examine our assumptions about *who* is caregiving and develop strategies for engaging and supporting all those who show up for care.

Palliative care research and practice is critically reflecting on inequities such as racism and discrimination that are embedded in its policies and practices.^30–34^ As part of this ‘equity turn’,^35^ attention is increasingly paid to the experiences of caregivers at the end of life whose disadvantage may be exacerbated by failing to attend to social and structural inequity.^1,9,36^ Research with people living in poverty, facing homelessness and housing vulnerability, and other structural oppressions like racism and discrimination is beginning to show that family caregiving may look different from normative notions of ‘family’ and ‘caregiving’ embedded in palliative care services and research.^36^ In research with people facing homelessness and vulnerable housing, three (diverse) groups of people emerge: (1) Unrelated people who may or may not be considered ‘family-like’ (e.g. street family, friends, neighbours)^36^; (2) Biological and/or legally related, hereafter referred to as bio-legal, family^15^ who may be fully or partially connected, estranged, or reunified at the end of life^36–39^; and (3) Community workers in health and social services who develop close and even ‘family-like’ relationships with people accessing services.^39–44^ It is clear from this body of research that people experiencing homelessness and unstable housing – defined in this essay as those living ‘rough’ or outside, in shelters or transitional housing, or at risk of homelessness^45^ – rely on those they are unrelated to for care, but these networks are neither well understood nor well supported by health systems and public policy.

In this critical essay, we aim to challenge embedded assumptions about family caregiving by white, Western systems (e.g. that of the nuclear family as carers) and focus on how the social determinants of health (SDOH; e.g. income and social protection, housing, education, food security) affect access to, and quality of, care at the end of life. More specifically, our analysis pays attention to what shapes the SDOH themselves including how racism, classism, heterosexism, and ableism become embedded and sustained in health and social institutions including palliative care.^33,46^ Our analysis is guided by the framework of structural vulnerability recognizing how social inequities embedded in systems are normalized and invisibilized, and function to privilege those at the top of social hierarchies, and exclude and disadvantage others.^46–49^ Our focus here is on highlighting these invisibilities to open up the space to reconsider caregiving in the context of inequities, unpacking dominant discourses found in the literature that have shaped the study of ‘family’. We begin by providing a brief discussion of the study of ‘family’ including the nuclear family standard and fictive kinship as an ‘alternative’ family form. Next, we focus in on fictive kinship in two diverse populations – (1) street-involved youth who form street families; and (2) older adults who access care beyond nuclear families – that challenge embedded assumptions and help set a foundation for thinking about family and caregiving in contexts of inequities. Drawing on short vignettes from our ongoing research programme, we then focus on equity-oriented approaches to ‘family’ caregiving at the end of life and conclude by offering ongoing questions, research and practice gaps, and suggestions for future research in this area.

## Conceptualizing family in contexts of inequity

### Nuclear family and ‘alternative’ family forms

Despite a wide variety of family and kinship models the world over, Western academia has largely focused on the nuclear family at the expense of expanded kinship in post-industrial societies.^16^ Indeed, the nuclear family – a heterosexual relationship between a male breadwinner and a female caregiver, and children cohabitating within a household – has set the standard for which research measures all other kinship relations.^15,50^ Western academics – largely originating from white, middle-class families – promoted the nuclear family as the ideal family structure to support the economy and society.^51,52^ Smith^50^ argued that the nuclear family as ‘normative’ has influenced how researchers have perceived ‘alternative’ family forms when they are happening. With far reaching consequences, nuclear family as a standard lead to policies and practices that exclude those that fall outside this standard while reinforcing the standard as real, true, and desired. Timmermans and Prickett^53^ argued that ‘standards embody social and cultural authority’ (p. 4) and operated outside laws to stratify society by determining who gets access to resources and who gets excluded from them. By continuing to study and attend to the nuclear family, expanded and different conceptualizations of family (e.g. extended family, community models) remain invisible, devalued, and under supported.

Social and demographic shifts, as well as movements promoting greater class, race, and gender diversity and justice, have challenged the normativity of the nuclear family and the abnormality of ‘alternative’ family forms.^15,16,52^ Studies of the family began to shift away from the nuclear family in the 1960s influenced by women moving into the workforce and feminist and civil rights movements.^52^ Furstenberg et al.^16^ highlighted how families have strayed from the nuclear structure through variations in marriage (e.g. divorce), reproductive processes (e.g. adoption), and families of choice. The social constructionist shift in family and kinship studies in the 1980s^16^ led to expanded definitions of family away from blood or legal relatedness or cohabitation (e.g. Nixon^48^). Increasingly, scholars studying family focus on how families define themselves. For instance, Galvin and Braithwaite^54^ described how families use communication within and outside the family to reinforce or reject family feeling and structure outside biological and legal relatedness. Attention is drawn to the ways that class, religion, community, geography, and other factors shape the definition and behaviours of family.^55^

### Fictive kinship

The study of ‘alternative’ family forms^16^ may be a later development in family and kinship studies, but the creation of ‘Fictive kin’ or people ‘like family’ but not related biology or legally^25,56^ has long been studied in anthropology and sociology. Fictive kinship recognizes the ways in which people hold extended definitions of kin beyond the people to whom they are related through blood, marriage, or formal adoption.^25,57^ Reviewing 600 articles that use the term fictive kin, Nelson^55,58^ concludes that fictive kin concepts are most often applied among African American and structurally oppressed groups. Carol Stack’s^59^ famous ethnography, for instance, examines fictive kinship as a resilient response by Black women living in the Midwestern United States in the 1970s for coping with poverty, racism, and to meet their needs for childcare, support, and material resources. Braithwaite et al.^23^ reviewed work on fictive/voluntary kin in contexts of structural oppression with African Americans, older adults, immigrants, working-class families, and youth concluding that possible functions of fictive kin include a, ‘sense of belonging, emotional closeness, protection and security, emotional support’ (p. 391). Nelson^58^ asserted that when researchers observe similar phenomena in white families, they use different terms such as ‘voluntary kin’.^23^ Debate remains as to whether extending kinship ties beyond bio-legal relatedness is more common among those facing social and structural inequity^16,60^ or whether the overrepresentation of these groups in research on fictive kinship has given off that impression.

Contending with diverse application, definition, and motivation for the formation of fictive kin, Nelson^60^ has proposed an ‘exploratory, descriptive typology’ (p. 261) of fictive kinship based on a variety of factors including the setting in which these relationships take place; type of individual; accomplishment of family including the benefits and costs; and other associated characteristics. Her three types are: (1) intentional kin, (2) ritual kin, and (3) situational kin. Intentional kin describes kinship relations similar to ‘chosen family’^61^ or ‘voluntary kin’.^23,24^ The second type is ‘ritual’ kin, which includes kinship formed through ceremony, which may maintain cultural ties or support the sharing of resources.^60^ Finally, ‘situational’ kin depicts kin relations that are not necessarily chosen, voluntary or intentional, or within ritual but rather, ‘occur within a special set of circumstances and is unlikely to involve the blood or legal family’ (p. 265),^60^ recognizing that the constrained choice present in situational kinship can lead to possibilities of exploitation and lack of consent in these relations.

The concept of fictive kinship is particularly relevant in the context of working with people who experience structural vulnerability. Here, we focus on two diverse groups – (1) homeless youth who form street families; and (2) older adults who access care beyond nuclear families – that may provide insights to our key focus on caregiving at the end of life in contexts of homelessness and housing vulnerability.

### Street-involved youth who form street families

While the concept of street family has been used to describe bio-legal families in the Global South who move from rural to urban settings as a result of urbanization and industrialization^62–64^ and queer youth of colour who take up public spaces in ‘Gaybourhoods’,^65^ we focus here on the application of the concept among young people experiencing homelessness, hereafter referred to as ‘street-involved youth’. One Canadian study^66^ conducted interviews with 480 youth (2/3 white, 1/3 men) aged 12–24 living in shelters, streets, or in temporary accommodation finding that 54% of their participants identified as being part of a ‘street family’ distinguished from other street-based relationships by the relationship type, roles, and responsibilities. Booth and Coveney^67^ conducted in-depth interviews with 15 homeless youth (9 women, 6 men) aged 15–23, in Adelaide, Australia. While the study initially focused on food insecurity among homeless young people, these authors identified street family as a potentially widespread phenomenon. Finally, Kennedy et al.^68^ examined the support networks of 130 youth (59% female, 28% ‘Aboriginal’) aged 14–18, with part or full time, street and informal economy involvement (e.g. panhandling, selling drugs), and identified both ‘off-street’ (bio-legal family) and ‘street-based’ relationships including friends and intimate partners and those that the youth considered family-like.

Studies on street-involved youth have shown how they form family-like relationships to fill unmet needs. These social, emotional, and material needs emerge from contexts of poverty and homelessness, food insecurity, and violence.^66,67^ Compared to non-family-like, street group relationships, street family is characterized by trust and reciprocity and significantly increased youth’s access to resources such as protection, money, and practical support for panhandling and the search for food and shelter.^66^ Street families function to share support for attainment of basic needs for food, protection, and shelter^67^ along with providing emotional support and a sense of love and belonging.^68^ Studies focused on street-involved youth illustrate how fictive kinship is a strategy to meet social, economic, and emotional needs (e.g. trust, reciprocity, love).

According to Nelson’s^60^ typology of fictive kinship, street family would fall under ‘situational’ kin – kin relations that are not necessarily chosen, voluntary or intentional, and where bio-legal family is absent or no longer has relevance in a person’s life. Situational kin is distinguished by the agency – or lack thereof – that the individual members have in establishing these fictive kin relations. Street family occurs under a further sub-category of ‘convenience kin’ in which relationships exist in contexts of marginality or among people who Nelson^60^ refers to as, ‘down and out regardless of race/ethnicity, age, or gender’ (p. 265). The author defines convenience kin as:

The fictive kinships that develop among unrelated individuals resemble a family of convenience: individuals separated from blood or legal kin (whether through their own choice or the actions of others) seek out other people on whom they can rely for both socioemotional and material support. (p. 268)

Convenience kin give their members a sense of identity and knowledge to adapt to shared, marginal situations, while filling material, emotional, and social support needs.^60^

Despite Nelson’s^60^ categorization of street-based relationships that are unlikely to involve bio-legal kin, studies of street-involved youth show variations in the involvement of bio-legal family members. Booth and Coveney,^67^ for instance, characterized street family relationships as an ‘obligation and duty to care’ (p. 50) but also described these relationships as more temporary in nature, often formed as a result of fleeing from violence and abuse from families of origin. This finding is similar to McCarthy et al.,^66^ who found that women, and those who had perpetuated and survived violence from bio-legal family members prior to street life, were more motivated to form street families as opposed to non-familial, street group relations. This is not to suggest that street-involved youth have no connections to bio-legal families. Kennedy et al.^68^ found that some youth had a great deal of support from street-based networks whilst maintaining relationships with bio-legal family and friends. These variations in family forms illustrate that, at least for youth, they are finding their sources of support through various means that extend beyond bio-legal family systems.

Together, research on street families in contexts of homelessness suggest that youth build support networks and relationships to meet their material, social, and emotional needs. While there is little evidence or theorizing related to how care needs get met in contexts of inequities for adults on the street, these learnings from research on street-involved youth are instructive for equity-oriented palliative care. Rising inequality, population ageing, neoliberal policies, and excessive mortality and morbidity among adults who are homeless, and experience other inequities means that adults experiencing homelessness are at particular risk when their health declines and when palliative care may be of benefit.^69^ The provision of palliative care, and its reliance on family caregivers, particularly for home- and community-based palliative care, raises questions about how caregiving occurs in these contexts. Insights from research on street-involved youth, above, and from studies in the social sciences, below, may provide insights into how caregiving in contexts of inequity might be considered. Considering the prevalence of accelerated ageing in the context of homelessness,^70–72^ this scholarship has the potential to offer important insights to a project on family and caregiving in contexts of inequity.

### Older adults who access care beyond nuclear families

A dearth of research on family caregiving in contexts of homelessness necessitates exploration into other contexts where expanded care networks appear. Braithwaite et al.^23,24^ argued that ‘voluntary kin’ networks become significant for older adults in need of care where they have outlived or become estranged from family or have no children of their own. While research in this area has focused on the vulnerability and isolation of these ‘kinless’ or ‘elder orphans’,^18,20^ recent research has sought to understand these expanded social networks and how they might be mobilized for care and help as adults age. For instance, Lowers et al.^28^ in a study of late-life disability and function of those 65 and older found that those ageing ‘solo’ were not, in fact, solo, but had diverse networks of unpaid caregivers including siblings, friends, neighbours, and bio-legal family members. Those ageing solo were also more likely to be women, Black non-Hispanic, and lower income than their married peers,^28^ indicating that social positioning and structural conditions may also play a role in expansive support networks. Scholars working in the area of gerontology and kinship provide insights into how and why older adults expand their networks for caregiving support as they age.

Providing an overview of why older adults might extend their kinship networks, Voorpostel^73^ introduced two related principles: substitution and replacement. While the ‘substitution’ principle alludes to choice in extending kinship in the absence or unavailability of family, the ‘replacement’ principle indicates the older adult’s establishment of fictive kinship as a consequence of rejection or estrangement from their family of origin. Substitution and replacement principles have been complicated and contested by other scholars studying fictive kinship among older adults. Allen et al.,^21^ for instance, found that the majority of their study participants converted non-kin to family-like roles indicating a high degree of fictive kin in their social networks. Fictive kinship, in this study, was a practice in which older adults largely participated, rather than *only* when bio-legal family were absent or unavailable, supporting the notion of fictive kin as supplemental to family that is biologically and/or legally related. While the fictive kin relations in their study offered support and companionship to the older adult participants, they did not preclude connection with related family members. Evidence of people expanding kinship networks even when bio-legal family is available speaks to the need for further exploration on how and why people are expanding kinship for care.

Like studies with street-involved youth, older adults are conceptualizing their relationship with unrelated kin in a variety of ways. Braithwaite et al.^23^ analyzed semi-structured interviews with 110 people (80% female, 88% Caucasian) in a university setting who self-identified as having ‘voluntary’ kin. Based on verbal descriptions of these kin, they found four types of voluntary kin relationships: (1) voluntary kin as a *substitute* for bio-legal family after death or estrangement; (2) voluntary kin as *supplemental* to bio-legal kin; (3) voluntary kin created around a specific context (e.g. for a fixed period of time, in a certain place); and (4) voluntary kin as extended family. Similar to the ‘replacement’ principle, Braithwaite et al.^23^ initially considered supplemental kin to result from *deficits* in bio-legal families. After a secondary analysis of these data, Braithwaite et al.^24^ revised this finding noting the variation in how people described voluntary kin in relation to their bio-legal kin. They presented an additional four structures to relations where both fictive and bio-legal kin were present in the care and support of older adults. These included: (1) intertwined; (2) limited; (3) separate; and (4) hostile. Where intertwined reflects a great deal of association between voluntary and bio-legal family, hostile represents aggression and ill will between the two family types. Beyond the fact that older adults are expanding their notion of family, this research highlights the importance of attending to the relationships between fictive kin and families of origin.

A convincing explanation for why older adults expand their kinship networks is to fill social, cognitive, emotional, and material support needs that are assumed to be met by family.^26,74^ In one of the earliest studies on fictive kinship in relation to caregiving, Johnson^25^ found that people aged 85 and older in the San Francisco Bay Area had fictive kin relationships or upgraded more distant kin to primary kin for caregiving and support. Allen et al.^21^ found that fictive kinship among older adults in their study was associated with the ‘process of securing social support and preparing themselves for handling future changes (e.g. losses) in their families’ (p. 1172). In a resource designed for nurses offering support to older adults at the end of life, Jordan-Marsh and Harden^26^ argued that ‘Adjusting one’s social network requires an active role where individuals and families bolster their circumstances under harsh conditions, such as chronic illness and disability or erosion of public assistance’ (p. 30). These authors suggest that social circumstances can compound the effects of ageing and illness creating the conditions for expanded kinship.

Evident in this brief overview of select studies in street homelessness and older adult settings is a variety of reasons for and ways to understand expanded family and care networks that challenge embedded assumptions in ‘family’ caregiving. The process of ageing and needing care and support can prompt older adults to expand their families and social networks outside biological and legal ties. When family is absent or unavailable, older adults substitute and replace them.^73^ However, even when family is available, there is evidence that older adults continue to create and engage with fictive kin.^21,23,24^ An equity-oriented palliative approach to care recognizes the strengths that exist in communities facing social and structural inequities including the care that is already happening.^69^ Accessing care and support beyond nuclear family is both an adaptive strategy for older adults^73,74^ and a practice that has existed for centuries among those excluded or devalued within mainstream family studies. In examining family caregiving in homelessness, there is a need to attend to cultural and ethnic difference such as Indigenous People experiencing homelessness^75^ who may consider kinship relations beyond nuclear family offering care to extended family and entire communities.^32,76–78^

## Emerging issues and future considerations in palliative care and ‘family’ caregiving in contexts of homelessness

Employing ethnographic, community-based, and participatory approaches, over a decade of research in Canada has illustrated complexities and challenges for people requiring and providing ‘family’ caregiving in contexts of inequities.^36,39,43,46,79–81^ Below, we offer short vignettes to ground a discussion of emerging issues and future considerations for research on ‘family’ caregiving in contexts of homelessness. While the details of these vignettes are fictitious, they offer problematics that illustrate the need to challenge assumptions in palliative care to better identify, engage, and support the caregiving that exists among friends and unrelated people, bio-legal family, and workers.

### Unrelated caregivers face similar health and social inequities

Tommy, a 55 year old homeless man calls the hospice unit to inquire about his street brother. When asked about the nature of their relationship, he replies, ‘Friend’. He is told that information can only be provided to family members or close friends approved by the patient. Given the phone communication, Tommy is hesitant to visit. Despite his worries, he panhandles the $5.00 required for the transit fare to visit his street brother forgoing his spot at the local shelter. Tommy arrives at hospice and is met with judgemental looks and questions about why he’s there. He’s told to leave his backpack and other belongings at the front door as the room is crowded. He leaves feeling unwelcome and decides not to return.

There is a dearth of studies focused on unrelated family caregiving in contexts of homelessness and unstable housing, but Stajduhar et al.^36^ suggested that people’s friends and neighbours who provide care are likely to *also* be facing health and social inequities (e.g. homelessness and unstable housing, financial problems, transportation barriers, racism) alongside those they support. If this is the case, then it is important to further examine how social and structural inequities bear down on people who are dying as well as their caregivers. Indeed, racism, classism, colonialism, ableism, and other inequities embedded in structures means that those facing social disadvantage are more likely to be homeless, while homelessness, itself, exacerbates social disadvantage.^45,75,82^ People experiencing homelessness have high rates of acute and chronic health conditions^83^ and face barriers to being diagnosed or receiving proper treatment and care when their medical conditions become serious.^39,84,85^ Studies show that serious illness management is complicated by homelessness and poverty as people experiencing health and social inequities are forced to navigate complex health systems while meeting their SDOH needs (e.g. shelter, food, transportation).^39,46,86^ When people experiencing homelessness access health and palliative care, they face stigma and discrimination including by a built environment, policies, and practices that exclude homeless people and their supports.^39,80,84,86^ In community settings, stigmatizing attitudes and organizational policy prevent access to care including the streets, shelters, and transitional and supportive housing.^39,42,81,87^ Identifying and supporting caregivers in contexts of homelessness will require attention to how the SDOH – and structural oppression – shape the ability for people to receive and offer care.

### Bio-legal family estrangement, reunification, and privileging

After a brain aneurism, David ends up in the ICU. His partner of 20 years, Loretta, who he refers to as his ‘wife’, is not called because there is no legal recognition nor documentation of their relationship. Hospital staff see that David has an ‘emergency contact’ on his electronic medical record. They call the number to find out it is his biological sister. Estranged for the last 10 years, David’s sister flies in from another region of the country to provide care and make decisions until he dies. Loretta fades into the background, unable to offer care or say goodbye. David’s ashes are sent back to his family in another region of the country while his partner grieves alone on the street.

Researchers working at the intersection of homelessness and end of life have identified a high degree of estrangement from bio-legal family,^41,44,88,89^ which sits in tension with assumptions embedded in palliative care about who most often provides care at the end of life. Studies on substitute decision-making – an important part of end of life care – have suggested variation in homeless people’s desire to involve bio-legal family. While homeless people have been found to appoint a bio-legal family member as their decision-maker,^37,38^ others have found a tendency towards naming friends or community housing and outreach workers^89^ or physicians.^90^ Bio-legal family can become involved at the end of life even when previously disconnected or estranged in life.^36,39^

Beyond the choice to reunite with or have bio-legal family become decision-makers, it is important to consider that health and social care systems may reinforce bio-legal conceptualizations of family^91^ despite people feeling differently. In a context where people’s family and support networks are not well understood or documented.^36,44^ bio-legal family may take on a more significant role at the end of life due to legislation and policy that privileges bio-legal over ‘chosen’ relationships and dominant discourses around family reunification at the end of life.^44^ Despite estrangement and separation, bio-legal family can gain power and legitimacy at end of life (through, for instance, decision-making legislation), which can cause significant distress for the caregivers who are left out.^36,43,44^ Workers and street families can be ignored and rejected as bio-legal family takes over end of life rituals, or ‘when rituals associated with dying and death are taken underground, hidden, or constructed in two different worlds’ (p. 249).^44^ In our studies, we have witnessed people living and working in the street community experience distress as they lose communication and connection with friends and family at the end of life.^39,42–44^ The minimal research on bio-legal family involvement at the end of life in contexts of homelessness illustrate a need to better understand these roles and relationships, and how bio-family may relate with other members of the caregiving network such as friends and street family including power dynamics between different groups.

### Community workers in between a formal and informal care role

Nancy, a woman with severe lung disease and a history of homelessness lives in transitional housing supported by a case manager named Georgia. Over the last year, Georgia has become very close to Nancy and learned that her family doesn’t visit her anymore and that she doesn’t have close friends. One day, the manager of the housing unit comes to Georgia to ask if Nancy’s level of care is too high for the facility. Georgia says ‘no’ because she does not want Nancy to be evicted and sent to hospital. Georgia begins doing more for Nancy ‘on the side’ such as taking Nancy’s clothes home to launder and cleaning up after Nancy’s incontinence. One day, Nancy has a fall and Georgia is wrought with guilt thinking that she should have told management earlier about Nancy’s decline. Unrecognized as an important part of Nancy’s caregiving network, Georgia is left to deal with her complicated grief and loss needs as Nancy is sent to hospital, then long term care, to spend her remaining days.

Workers have been found to fill gaps left by estranged family members and small support networks^39,42–44^ producing blurred lines between formal and informal care. Community workers have been seen going ‘above and beyond’ their job scopes and roles to offer care to people with serious illness when others are not available to do so^39–44,92^ and sometimes becoming ‘defacto’ family members.^39,41,81^ In their research with staff members providing medical and social support to homeless people in a Swedish support home, Håkanson et al.^41^ identified how staff become ‘professional family’ (p. 1260) forming trusting, family-like relationships, and environments. These relationships were created by replacing bio-legal family members, advocating for their patients in health settings, and through everyday interactions (e.g. going on walks, watching TV). Similarly, in a report on Ambrose Place,^92^ a facility in Canada offering culturally appropriate housing and support services to Indigenous People, it is described how both staff and residents feel like they belong to a family. Residents and staff were found to use family-like terms to describe their co-workers and co-residents and reported a felt sense of love, belonging, respect, and care.

Variations exist in the described impact of these blurred informal/formal care roles played by workers. Some highlight how ‘family-like’ roles have the potential to cause conflict between workers and the people they are paid to serve and can illustrate a lack of professionalism and violation of personal and organizational boundaries,^41^ and contribute to staff burnout.^40^ Others push back against the notion that loving and caring for their residents like family is unprofessional^92^ and suggest the need for more supportive organizational policies to recognize the impacts of inequities on caring, dying, death, and bereavement.^80,93^ As it relates to palliative care, the close relationships that people inevitably develop with the people they are supporting including those who are members of the communities in which they serve – in relation to Indigenous or peer workers – impacts their grief and bereavement experiences. Studies have highlighted how unjust and unsupported deaths result in deep grief for those working in contexts of homelessness and the toxic drug supply crisis.^80,93,94^ These studies demonstrate how the context of life and death experiences (i.e. shortened lives because of homelessness, discrimination, criminalization, and racism) is an essential part of understanding caregiver experiences.^95^ There is a need to better understand the informal/formal caregiver role that many workers find themselves in, and develop organizational responses that value and attend to this important care work.

### Areas of future research and exploration

While we have thus far largely discussed family and people considered ‘like’ family (e.g. fictive kin), studies on street-involved youth urge us to consider the care and support offered by people who are not considered family at all. Indeed, Kennedy et al.^69^ found that youth did not consider all their street relations to be family-like and characterized these non-family-like relations as less stable and shorter in duration. Yet, even in these non-family-like relations, youth described important companionship and instrumental support (e.g. shared resources, protection). Family abolitionists^96^ argue that the organization of society into families encourages invisible and unrecognized work, privatizing care, and relieves the state from its duties to care for its citizens. Just as Smith^50^ identified how researchers’ cultural bias of nuclear family enables them to ‘see’ nuclear family at the expense of other family and kinship models, we might also disassociate family from the concept of caregiving to see how caregiving is happening outside family and family-like concepts.

Going forward, there is a need to investigate how people experiencing homelessness alongside other health and social inequities define and access care from unrelated caregivers. At the same time, we must account for the standard of the nuclear family and how bio-legal privileging may create inequities in an end of life context where people interface with legal and financial systems alongside health and social systems. Timmermans and Prickett^53^ reveal how people’s requirement for state resources can force them to fit into the standard of the nuclear family as well as reclaim other family formations. In the future, we may look to research in ‘non-kin’ caregiving^22,97–99^; research with sexual and gender minority older adults^100–102^; Indigenous worldview and approaches around family and kin at the end of life^32,76,78^; and Public Health Palliative Care and Compassionate Cities movements that emphasize the role of friends, neighbours and neighbourhoods, and community associations in responding to death, dying, care, and grief^103,104^ to help to challenge our assumptions of the nuclear family as caregivers and to inform recommendations for engagement and support of all people who show up for care.

## Conclusion

In a world that is becoming more inequitable, understanding and reducing health disparities is a key priority for palliative care. This essay has demonstrated that bio-legal assumptions and privileging may be yet one more inequity in palliative care to address and overcome. This essay has focused in on populations facing homelessness and housing vulnerability, but changing families and growing inequality suggests the potential broad applicability of this work for our collective future. Palliative care is one of the areas of the Western healthcare system that explicitly attends to the person with serious illness and their family and caregivers understanding deeply how the suffering and joys of one group impacts the other. If there is a place in the current health system that can truly make space for caregiving – in all the ways it happens – it is, and should be, palliative care.
